# Hyperventilation: A Possible Explanation for Long-Lasting Exercise Intolerance in Mild COVID-19 Survivors?

**DOI:** 10.3389/fphys.2020.614590

**Published:** 2021-01-18

**Authors:** Justina Motiejunaite, Pauline Balagny, Florence Arnoult, Laurence Mangin, Catherine Bancal, Marie-Pia d’Ortho, Justine Frija-Masson

**Affiliations:** ^1^Service de Physiologie-Explorations Fonctionnelles, FHU APOLLO, Assistance Publique Hôpitaux de Paris, Hôpital Bichat-Claude Bernard, Paris, France; ^2^INSERM, UMR 1141 NeuroDiderot, Université de Paris, Paris, France; ^3^INSERM, UMS 011, Population-based Epidemiological Cohorts Unit, Villejuif, France; ^4^UMR 7057, CNRS, Laboratoire Matière et Système Complexes, Paris, France

**Keywords:** COVID-19, hyperventilation syndrome, dyspnea, cardiopulmonary exercise testing, SARS-CoV-2, exercise hyperventilation, persisting symptoms

## Abstract

Since the outbreak of the coronavirus (COVID-19) pandemic, most attention has focused on containing transmission and addressing the surge of critically ill patients in acute care settings. As we enter the second phase of the pandemic, emphasis must evolve to post-acute care of COVID-19 survivors. Persisting cardiorespiratory symptoms have been reported at several months after the onset of the infection. Information is lacking on the pathophysiology of exercise intolerance after COVID-19. Previous outbreaks of coronaviruses have been associated with persistent dyspnea, muscle weakness, fatigue and reduced quality of life. The extent of Covid-19 sequelae remains to be evaluated, but persisting cardiorespiratory symptoms in COVID-19 survivors can be described as two distinct entities. The first type of post-Covid symptoms are directly related to organ injury in the acute phase, or the complications of treatment. The second type of persisting symptoms can affect patients even with mild initial disease presentation without evidence of organ damage. The mechanisms are still poorly qualified to date. There is a lack of correlation between initial symptom severity and residual symptoms at exertion. We report exercise hyperventilation as a major limiting factor in COVID-19 survivors. The origin of this hyperventilation may be related to an abnormality of ventilatory control, by either hyperactivity of activator systems (automatic and cortical ventilatory control, peripheral afferents, and sensory cortex) or failure of inhibitory systems (endorphins) in the aftermath of pulmonary infection. Hyperventilation-induced hypocapnia can cause a multitude of extremely disabling symptoms such as dyspnea, tachycardia, chest pain, fatigue, dizziness and syncope at exertion.

## Introduction

The infection with severe respiratory syndrome coronavirus 2 (SARS-CoV-2) was declared a pandemic on March 11, 2020. As of November 2020, the novel coronavirus, hereafter referred to as COVID-19, has affected more than 60 million people worldwide (Center, 2020; Simpson and Robinson, 2020). For the majority (81%), infection with COVID-19 manifests as a mild disease. Fever (88.7%), cough (57.6%), and dyspnea (45.6%) were the most commonly reported symptoms in a recent systematic review (Rodriguez-Morales et al., 2020). However, for a significant minority, and particularly those older than 65 years and with comorbidities, the infection requires management in an intensive care unit due to acute respiratory distress syndrome (ARDS) (Pascarella et al., 2020). Since the outbreak of the pandemic, most attention has focused on containing transmission and managing the wave of critically ill patients in acute care settings. As we enter the second phase of the pandemic, emphasis must evolve to post-acute care of COVID-19 survivors. For these patients, having defeated the virus is just the beginning of an unknown recovery path. There are increasing reports of persisting and recurrent symptoms at several months after the onset of the infection (Carfi et al., 2020; Goërtz et al., 2020), dyspnea at exertion being one of the most common complaints. The extent and severity of the lasting sequelae remain to be evaluated, but persisting cardiorespiratory symptoms in COVID-19 survivors can be described as two distinct entities. The first type of post-Covid consequences are directly related to organ damage in the acute phase, either due to the viral infection and the ensuing inflammatory response, or the complications of treatment in the intensive care setting (Li et al., 2006; Zhou et al., 2014; Mart and Ware, 2020). The persisting cardiorespiratory symptoms related to cardiac and lung injury in COVID-19 will be summarized below. The second type of persisting symptoms can affect patients even with mild initial disease presentation without evidence of severe organ damage. Sustained fatigue and exercise intolerance are the most frequent complaints in patients not requiring hospitalization (Goërtz et al., 2020; Tenforde et al., 2020). In these patients, there is a striking lack of correlation between initial symptom severity and residual symptoms at exertion. The mechanisms behind post-Covid exercise intolerance are most likely complex and still poorly qualified to date. In this article, we aimed to review the available evidence on persisting exercise intolerance experienced by COVID-19 survivors. We also report exercise hyperventilation as a major limiting factor that can cause a multitude of extremely disabling symptoms such as dyspnea, tachycardia, chest pain, fatigue, dizziness, and syncope at exertion. To our knowledge, this is the first report of a possible explanation for prolonged exercise intolerance in long-lasting COVID-19.

## Persisting Cariorespiratory Symptoms in Coronavirus Survivors: Current State of Knowledge

Around 20% of patients with severe COVID-19 require in-hospital management (Simpson and Robinson, 2020). For the patients presenting with severe initial illness, the long-term consequences are yet to be elucidated. However, emerging studies show evidence of persistent cardiorespiratory symptoms months after hospital discharge. Weerahandi et al. (2020) studied severe COVID-19 patients who required at least 6 l/min of oxygen during hospital stay. At one-month follow-up, 74% of participants reported shortness of breath. Carfi et al. (2020) also assessed persistent symptoms in patients discharged from the hospital. At 2 months follow-up, half of the patients reported persistent fatigue, whereas dyspnea (43%) and chest pain (22%) were also highly prevalent. These findings are in line with the studies by Wong et al. (2020) and Garrigues et al. (2020), both of which found nearly half of the patients complaining of breathlessness at 3 months after hospital discharge.

The persistence of cardiorespiratory symptoms in survivors of severe COVID-19 can be partly explained by the pathophysiology of organ damage during the initial phase of the disease. The SARS-CoV-2 virus predominantly affects the respiratory system, although other organ systems can be compromised as well. The virus uses angiotensin-converting enzyme-2 (ACE2) receptors in pneumocytes of the epithelial alveolar lining to infect the host, thus causing lung injury (Varga et al., 2020). Diffuse alveolar damage was showed by several post-mortem studies (Carsana et al., 2020; Schaller et al., 2020), leading to hypotheses of residual pulmonary function impairment at long term. These hypotheses are supported by the available evidence on previous coronavirus outbreaks. A recent meta-analysis (Ahmed et al., 2020) of studies on SARS-CoV and MERS-CoV reported that approximately one third of hospitalized patients had persisting lung abnormalities after their acute illness. Prospective studies on SARS-CoV, MERS-CoV as well as short-term follow-up studies on the current SARS-CoV-2 epidemic are summarized in Table 1. These studies demonstrate mostly a mild pulmonary function impairment and the functional disability appears out of proportion to the degree of lung function impairment. For example, a 2005 study on SARS-CoV by Hui et al. (2005) found that the exercise capacity 6 months after disease onset was considerably lower than that of normal controls in the same age groups. However, significant impairment of surface area gas exchange was only found in 15% of the patients. Along with Hui’ study, Ong et al. (2004) performed cardiopulmonary exercises testing in 46 SARS-CoV patients at 3 months after hospital discharge. Half of the patients had abnormalities in the pulmonary function tests, but the impairment was mild in almost all cases. However, 41% of the patients had impairment of exercise capacity not due to ventilatory limitation. The discordance of the results of pulmonary function and exercise testing has been attributed to several factors, such as physical deconditioning, muscle weakness and poor motivation. The recent data in SARS-CoV-2 by Arnold et al. (2020) demonstrates that even 74% of survivors complained of persistent breathlessness and excessive fatigue at 3 months after hospital discharge. Yet abnormal pulmonary function was found in only 10% of the patients. Major limitations have to be taken into account when considering available evidence on long-term pulmonary function abnormalities. The majority of information is derived from single-center studies with a small sample of patients and a relatively short-term follow-up. However, most of the data support the idea that persistent cardiorespiratory symptoms in COVID-19 survivors cannot be accounted for by impairment of pulmonary function alone.

**TABLE 1:** prospective studies on residual symptoms, pulmonary function impairment and exercise intolerance following SARS-CoV, MERS-CoV and SARS-CoV-2 viral infections.

	Study	Year	Number of patients	Assessment modality	Duration of follow-up	Main findings
SARS-CoV	Zhang et al., 2020	2018	71	PFT	15 years	No data on residual symptoms Mildly reduced D_L_CO in 38% of patients Reduced FEV_1_/FVC ratio in patients with residual chest CT abnormalities
	Ngai et al., 2010	2010	123	PFT and 6MWD	2 years	Lower quality of life compared to normal controls Persistent impairment of D_L_CO in 52% of survivors Reduced 6MWD compared to controls
	Li et al., 2006	2006	36	PFT and 6MWD	1 year	Reduced quality of life in patients older than 40 years Mild impairment of D_L_CO with near normal PFT Near-normal 6MWD
	Hui et al., 2005	2005	110	PFT and 6MWD	6 months	Impairment of health-related quality of life D_L_CO impairment in 15.5% of survivors Reduced 6MWD compared to controls
	Ong et al., 2004	2004	46	PFT and CPET	3 months	Shortness of breath in 50% of patients Abnormalities on PFT in 50% of patients Reduced maximum aerobic capacity in 41% of patients
MERS-CoV	Park et al., 2018	2018	73	PFT and 6MWD	1 year	No data on residual symptoms 8% of patients had lung function parameters <80% of predicted values Impairment of D_L_CO in 34% of all patients Preserved 6MWD
SARS-CoV-2	Zhao et al., 2020	2020	55	PFT	3 months	Persisting symptoms in 60% of patients Lung function abnormalities in 25%
	Clavario et al., 2020	2020	150	PFT and CPET	3 months	Normal PFT Functional capacity limitation in 50% of patients, mainly explained by muscular impairment
	Raman et al., 2020	2020	58	PFT, 6MWD, and CPET	2–3 months	Persistent breathlessness in 64% of patients Shorter 6MWD compared to controls Reduced exercise capacity in 55% of patients
	Arnold et al., 2020	2020	163	PFT	2–3 months	Persistent breathlessness and excessive fatigue in 74% of patients Restrictive syndrome in 10%
	van Gassel et al., 2020	2020	48	PFT and 6MWD	2 months	No data on persistent symptoms Diminished total lung capacity and diffusion capacity in half of the participants 6MWD result was 82% of predicted distance
	Frija-Masson et al., 2020	2020	50	PFT	1 month	Abnormal lung function in half of the patients, without a clear relationship with CCT findings
	Huang Y. et al., 2020	2020	57	PFT and 6MWD	1 month	Impaired D_L_CO and lung imaging abnormalities in 50% of patients, in relation to initial disease severity
	Mo et al., 2020	2020	110	PFT	1 month	Anomalies in D_L_CO in 47% of patients, in relation to pneumonia severity

*CCT, chest computed tomography; PFT, pulmonary function tests; 6MWD, 6-min walking distance; CPET, cardiopulmonary exercise testing; MRI, magnetic resonance imaging; D_L_CO, diffusing capacity of the lung for carbon monoxide; FEV_1_/FVC, forced expiratory volume in 1 s to forced vital capacity. As the COVID-19 outbreak continues to evolve and the scientific evidence rapidly expands, the information provided in this table is only current as of the date of submission.*

Multisystem involvement is the key pathophysiological feature of SARS-CoV-2 infection and can help explain the heterogeneous symptoms experienced by patients, as well as the complex mechanisms of long-lasting exercise intolerance. As described above, the virus binds to ACE2 receptors, which are present not only in the lungs, but are widely distributed in endothelial cells. Endothelial injury recruits inflammatory leukocytes, and contributes to tissue damage and cytokine release, as well as thrombosis and disseminated intravascular coagulation (Evans et al., 2020). Autopsy studies (Varga et al., 2020) show severe endothelial injury that is associated with the presence of intracellular virus. Widespread thrombosis with microangiopathy were also described (Ackermann et al., 2020). It has been postulated that endothelial injury and microangiopathy might be the reasons behind autonomic dysfunction, which is compatible with residual symptoms such as fatigue, palpitations and chest pain, among others. Cardiac injury has also been documented in patients hospitalized with moderate or severe COVID-19 (Clerkin et al., 2020; Driggin et al., 2020; Huang C. et al., 2020). Inflammatory myocarditis is frequently diagnosed in severe COVID-19 patients and may later lead to left ventricular failure. Inflammation induced pro-coagulatory state might lead to type 1 myocardial infarction (MI) due to plaque rupture or intracoronary thrombosis (Chieffo et al., 2020; Clerkin et al., 2020). Type 2 MI (myocardial injury in the absence of obstructive coronary artery disease) might be induced by prolonged hypoxia. Right ventricular failure secondary to acute pulmonary embolisms is also possible. Myocardial injury can persist after the acute phase and clinically manifest as dyspnea or chest pain at exertion. Therefore, appropriate follow-up to detect these complications is warranted. Two recent studies with cardiac magnetic resonance imaging (MRI) (Huang L. et al., 2020; Puntmann et al., 2020) showed ongoing cardiac involvement in a majority of patients months after a COVID-19 diagnosis. Another study by Raman et al. (2020) describes persistent MRI abnormalities seen in the lungs (60%) and the heart (26%) of COVID-19 patients at 2–3 months after hospital discharge, in relation to ongoing symptoms of breathlessness and excessive fatigue (in 64 and 55% of patients respectively). In the same study, cardiopulmonary exercise testing showed that 55% of patients had peak oxygen uptake (VO_2_ max) values lower than 80% of the predicted. In contrast, only 12% of patients had impaired pulmonary function tests.

Persisting cardiorespiratory symptoms may be in part related to initial disease severity and residual organ damage. In critically ill patients, acute respiratory distress (ARDS) – related consequences have been described previously (Desai et al., 1999; Mart and Ware, 2020) and are not specific to COVID-19. Other ICU-related complications such as ventilator-induced lung injury or critical illness-associated poly-neuropathy are well established (Zhou et al., 2014; Beitler et al., 2016) and are out of the scope of this article. The routine administration of high dose steroids to many patients with ARDS might lead to steroid myopathy and muscle wasting, resulting in residual exercise intolerance and excessive fatigue.

Most of the studies that have reported on sequelae of COVID-19 included participants whose illness was severe enough to require hospitalization. However, the majority of patients with COVID-19 are managed in outpatient settings. Long term outcomes might not be comparable due to multiple factors: varying degree of organ damage, different treatment and distinct patient demographics, as many patients admitted to hospital with COVID-19 are older, have more comorbidities and frailty. Despite mild initial presentation, emerging evidence indicates that it might take weeks for resolution of symptoms and return to usual health. Tenforde et al. (2020) reported that out of 274 patients tested for COVID-19 in an outpatient setting, one third complained of fatigue and dyspnea 2 weeks after symptom onset. Even among young adults aged 18–34 years with no chronic medical conditions, nearly one in five reported that they had not returned to their usual state of health. Thus it seems that the persistence of symptoms might not necessarily be related to patient age or initial clinical severity of the disease.

These findings were further confirmed by Goërtz et al. (2020), who performed a survey in ambulatory COVID-19 patients at 3 months after symptom onset. They showed that the majority of patients were still symptomatic, and reported a multitude of symptoms, ranging from cough, sore throat, muscle pain, dizziness, chest tightness, palpitations, weight loss, etc. Fatigue and dyspnea were the two most prevalent symptoms, described by 87 and 71% of patients respectively. These findings are especially alarming for such a young population (median age of 47 years) mostly without serious comorbidities and normal physical examination.

To date, the etiology of these heterogeneous long-Covid symptoms is poorly understood. It is possible that endothelial injury might play a role in the persistence of dysautonomic symptoms in COVID-19 survivors. Davido et al. (2020) described their experience working with outpatients who experienced mostly mild symptoms attributable to COVID-19. Subsequently they observed multiple persistent symptoms, especially intense fatigue, shortness of breath, chest tightness and tachycardia. Authors suggest that these symptoms are compatible with dysautonomia due to microangiopathy and endothelial injury. Miglis et al. (2020a, b) also described a subset of COVID-19 survivors presenting symptoms of autonomic dysfunction such as orthostatic intolerance and postural orthostatic tachycardia. Such symptoms are frequently reported after other viral infections and might be related to gastrointestinal fluid loss, prolonged bed rest and deconditioning of the cardiovascular system. However, further research is needed to further characterize the dysautonomic syndromes in COVID-19 survivors.

In contrast to hospitalized COVID-19 patients, conventional risk factors such as age and the presence of comorbidities do not seem to have an impact on the duration and severity of persistent symptoms. A study by O’Keefe et al. (2020) analyzed 273 non-hospitalized patients recovering from COVID-19. Interestingly, the authors found no correlation between symptom duration and patient factors such as age or comorbidities.

All in all, the pathophysiology of persistent cardiorespiratory symptoms in COVID-19 outpatients are not clearly understood to date. Even though these symptoms seem to be benign, their importance should not be underestimated. Persisting exercise intolerance can result in worsened quality of life, inability to return to work and increased use of health care systems, constituting a worldwide public health problem.

## Hyperventilation as a Possible Cause of Persisting Exercise Intolerance

As a French Reference Center for Infectious Diseases, Bichat hospital has had an important number of COVID-19 patients, treated both in and out of hospital settings. All patients are systematically offered follow-up and undergo a thorough cardio-pulmonary exploration, including pulmonary function tests (PFT), a chest CT scan, a trans-thoracic echocardiogram, and cardiopulmonary exercise testing (CPET). Ethics committee approvals were obtained according to local requirements.

We report a case series of eight patients (seven women and one man) with important exertional dyspnea at 3 months after the onset of COVID-19 symptoms. All patients were aged 31–73 [median age 39 years (interquartile range 33–49)] and had no previous medical history (notably, no chronic cardiovascular or pulmonary diseases). All of the patients had a relatively mild course of COVID-19 and received ambulatory treatment without indications for hospitalization or oxygen therapy. Nijmegen test was available in five patients and median score was 35 (27–38). At 3 months after the symptom onset, all patients had near-normal pulmonary function tests and normal chest CT scans. Transthoracic echocardiogram showed preserved left ventricular ejection fraction as well as the absence of pulmonary hypertension (see Table 2). During CPET, dyspnea, palpitations, and dizziness were reproduced in all patients. For two patients CPET had to be interrupted due to syncope at exertion. All of the patients showed a significant impairment of exercise capacity: seven out of eight were incapable of reaching 100% of predicted workload and none of the patients reached their predicted VO_2_ max values. An elevated VE/VCO_2_ ratio was observed in five patients, suggesting exercise hyperventilation. All patients increased their ventilation and their tidal volume as soon as the effort started. However, we were not able to define the generic ventilatory profile since two patients increased their respiratory rate (RR) only after the aerobic threshold and one patient had no significant increase in RR. Unfortunately, we were not able to collect data on inspiratory capacity. Arterial blood gasses were also analyzed at rest as well as at maximum effort. The apparition of respiratory alkalosis with low arterial CO_2_ levels and significant base excess at exertion was observed in three patients. We hypothesized that hyperventilation-induced hypocapnia following COVID-19 infection and prolonged bed rest might be responsible for a multitude of extremely disabling symptoms such as dyspnea, tachycardia, chest pain, fatigue, dizziness and syncope at exertion. Indeed, clinical follow-up of patients showed progressive resolution of symptoms. Interestingly, most patients described paroxysmal dyspnea that could also occur at rest, which could indicate a wider breathing dysfunction. Normality of PFT is in accordance with our hypothesis that alteration of lung function does not solely account for residual symptoms.

**TABLE 2:** Characteristics of patients presenting with hyperventilation syndrome in the aftermath of COVID-19, and findings of cardiopulmonary exercise testing.

Patient No.	1	2	3	4	5	6	7	8
Gender	F	F	F	F	F	F	F	M
Age, years	73	32	35	48	49	31	35	39
BMI, kg/m2	25.0	18.7	20.4	21.5	26.2	18.0	30.1	33.3
**Cardiorespiratory parameters at rest**								
RR at rest, rpm	52	16	31	19	22	31	10	24
Tidal volume at rest, L	1.14	0.45	0.40	0.72	0.60	0.36	1.37	0.83
FEV_1,_%	85	88	116	107	109	75	101	88
FEV_1_/VC	0.68	0.84	0.81	0.80	0.85	0.89	0.80	0.7
TLC,%	103	97	113	90	89	85	103	73
D_L_CO,%	79	83	82		62	67	60	68
HR at rest, bpm	121	82	88	104	73	101	78	107
BP at rest, mmHg	110/70	152/99	101/73	119/87	93/70	89/63	106/63	127/88
LVEF,%	63	70	75	68	68	56	64	66
**Cardiorespiratory parameters during exercise**								
Load reached (W)	47	113	147	100	90	78	70	50
% of predicted load	82	87	119	96	95	63	54	22
% of target heart rate	84	98	82	90	97	87	59	67
Respiratory rate at peak exercise	44	45	46	25	61	48	32	38
Tidal volume at peak exercise, L	0.87	1.08	1.48	1.75	1.26	0.85	1.54	1.50
Minute ventilation at peak exercise, L/min	45	48	69	45	75	44	52	55
Peak respiratory exchange ratio	1.08	1.29	1.17	1.19	1.22	1.06	1.17	1.24
Breathing reserve,%	48	59	51	77	23	53	58	46
VO_2_ max, ml/kg/min	10.4	21.2	28.6	17.6	22.8	17.3	11	8.2
% of predicted VO_2_ max	62	64	93	70	96	47	49	29
VE/VCO_2_	49	32	37	34	44	48	46	51
Symptoms at exertion	dyspnea	dyspnea, palpitations, chest pain	dyspnea, dizziness, tingling	dyspnea	dyspnea	dyspnea, dizziness, syncope	dyspnea	dyspnea, chest pain, syncope
**Arterial blood gasses at rest and at peak exercise**								
pH at rest	7.55	7.41	7.45	7.47	7.46	7.45	7.44	7.41
pH at exertion	7.42	7.33	7.38	7.41	7.42	7.47	7.47	7.51
PaO2 at rest, mmHg	90	94	100	98	99	100	81	91
PaO2 at exertion, mmHg	92	92	102	104	102	120	116	117
PaCO2 at rest, mmHg	23	39	36	34	32	30	32	39
PaCO2 at exertion, mmHg	31	37	35	32	33	23	27	26
HCO3 at rest, mmol/l	20	25	25	25	23	21	22	25
HCO3 at exertion, mmol/l	20	19	20	20	22	16	19	21
Nijmegen score		20	34	37			40	35

*BMI, body mass index; RR, respiratory rate; HR, heart rate; BP, blood pressure; LVEF, left ventricular ejection fraction; FEV_1_, forced expiratory volume in 1 s; FEV_1_/VC, the ratio of forced expiratory volume on vital capacity; TLC, total lung capacity; D_L_CO, diffusing capacity for carbon monoxide. All pulmonary function values are presented as predicted percentage considering age, sex, height, body weight, and race.*

Exercise hyperventilation is a condition defined by inappropriate alveolar hyperventilation, in regards to metabolic needs and mechanical stress in the body (Brat et al., 2019). It generally affects women more frequently than men, especially between the ages of 15 and 55 (Lum, 1975). The variety of symptoms experienced by the patient might take them to a wide range of specialist consultations, numerous investigations and inappropriate treatment.

The origin of this hyperventilation is unknown. The hypothesis of an abnormality of ventilatory control has been proposed, by either a stimulation of activator systems (automatic and cortical ventilatory control, peripheral afferents, and sensory cortex) or a suppression of inhibitory systems (endorphins) in the aftermath of a pulmonary infection (Gardner, 1994). Deciphering the mechanisms underlying this hyperventilation would require to examine chemo responsiveness by hypercapnic ventilatory challenge in patients recovering from COVID-19 and compare patients with and without hyperventilation.

Various forms of primary lung disease can influence the ventilatory control mechanisms and alter the respiratory center output. Hypoxemia-induced activation of peripheral chemoreceptors or stimulation of receptors by diseases affecting the airways or pulmonary interstitium can induce the respiratory center to increase its output, resulting in respiratory alkalosis. Similarly, patients recovering from acute pulmonary embolism, pneumonia, or chronic interstitial lung disease often hyperventilate, probably as a result of stimulation of one or more types of intrathoracic receptors, with or without the additional ventilatory stimulus induced by hypoxemia (Jack et al., 2003).

Even though the mechanisms of hyperventilation are not quite clear, the consequences of alveolar hyperventilation are well known (Laffey and Kavanagh, 2002). The most important physiological consequence of alveolar hyperventilation is the decrease in depolarization threshold of cell membranes. In case of respiratory alkalosis, H^+^ ions do not participate in membrane potential, and are transported out of the cell to decrease the blood pH, whereas K^+^ ions are transported into the cell. A relative excess of positive ions inside the membrane increases its potential, therefore decreasing the depolarization threshold. Neuronal hyperexcitability causes the activation of autonomous nervous system which in turn is responsible for neurovegetative symptoms described in hyperventilation syndrome. Muscular hyperexcitability results in increased muscular tone and arterial vasoconstriction due to smooth muscle cell contraction in the arterial wall. Resulting hypoperfusion of different organs might cause various symptoms related to ischemia. Hyperventilation related symptoms can range from dyspnea, palpitations, chest pain, muscle cramps, syncope to paresthesia, dizziness, headache, abdominal pain, nausea, fatigue, and anxiety.

There is no gold standard for the diagnosis of hyperventilation syndrome. Diagnostic tools include arterial blood gas measurement and various provocation tests in order to reproduce the symptoms. The Nijmegen Questionnaire (van Dixhoorn and Duivenvoorden, 1985) may be used as a screening instrument for early detection of hyperventilation syndrome, as well as an aid in diagnosis and therapy planning (with a sensibility of 91% and specificity 95%). Treatment options include respiratory physiotherapy with an experienced respiratory therapist, focusing on patient education and various techniques to control breathing. Fortunately, spontaneous resolution is common, especially with the gradual resumption of physical activity.

## Conclusion

Persisting cardiorespiratory symptoms are rather common at several months after the onset of the COVID-19 infection. Currently, data is lacking on the pathophysiology of this post-COVID entity. In patients with severe initial disease presentation, persisting symptoms might be directly related to target organ damage as well as treatment complications. New studies have emerged describing the post-COVID syndrome even in patients with mild initial presentation, without a clear relationship with age and comorbidities. Persistent cardiorespiratory symptoms in these patients are most likely benign and unrelated to long-term organ damage. Muscle deconditioning, dysautonomia and exercise hyperventilation might partly explain the disabling symptoms in COVID-19 survivors. However, more studies are needed to further clarify the mechanisms behind this prolonged path to recovery.

## Author Contributions

JM, PB, FA, LM, CB, and JF-M performed the cardiopulmonary function tests. JM and PB performed a literature review. JM and JF-M drafted the manuscript. PB, FA, LM, CB, and M-Pd’O contributed significantly to manuscript correction and finalization. All authors contributed to the article and approved the submitted version.

## Conflict of Interest

The authors declare that the research was conducted in the absence of any commercial or financial relationships that could be construed as a potential conflict of interest.

## References

[B1] Ackermann M. Verleden S. E. Kuehnel M. Haverich A. Welte T. Laenger F. (2020). Pulmonary vascular endothelialitis, thrombosis, and angiogenesis in COVID-19. *N. Eng. J. Med.* 383 120–128. 10.1056/nejmoa2015432 PMC7412750 32437596

[B2] Ahmed H. Patel K. Greenwood D. Halpin S. Lewthwaite P. Salawu A. (2020). Long-term clinical outcomes in survivors of coronavirus outbreaks after hospitalization or icu admission: a systematic review and meta-analysis of follow-up studies. *medRxiv [preprint]* 10.1101/2020.04.16.20067975 32449782

[B3] Arnold D. T. Hamilton F. W. Milne A. Morley A. Viner J. Attwood M. (2020). Patient outcomes after hospitalisation with COVID-19 and implications for follow-up; results from a prospective UK cohort. *medRxiv [preprint]* 10.1101/2020.08.12.20173526 PMC7716340 33273026

[B4] Beitler J. R. Malhotra A. Thompson B. T. (2016). Ventilator-induced lung injury. *Clin. Chest Med.* 37 633–646. 10.1016/j.ccm.2016.07.004 27842744 PMC5131805

[B5] Brat K. Stastna N. Merta Z. Olson L. J. Johnson B. D. Cundrle I. Jr. (2019). Cardiopulmonary exercise testing for identification of patients with hyperventilation syndrome. *PLoS One* 14:e0215997. 10.1371/journal.pone.0215997 31013331 PMC6478351

[B6] Carfi A. Bernabei R. Landi F. Gemelli Against C.-P.-A. C. S. G. (2020). Persistent symptoms in patients after acute COVID-19. *JAMA* 324 603–605. 10.1001/jama.2020.12603 32644129 PMC7349096

[B7] Carsana L. Sonzogni A. Nasr A. Rossi R. S. Pellegrinelli A. Zerbi P. (2020). Pulmonary post-mortem findings in a series of COVID-19 cases from northern Italy: a two-centre descriptive study. *Lancet Infect. Dis.* 20 1135–1140. 10.1016/s1473-3099(20)30434-5 32526193 PMC7279758

[B8] Center J. H. C. R. (2020). *COVID-19 Map - Johns Hopkins Coronavirus Resource Center. [online].* Available online at: https://coronavirus.jhu.edu/map.html (accessed November 26, 2020).

[B9] Chieffo A. Stefanini G. G. Price S. Barbato E. Tarantini G. Karam N. (2020). EAPCI position statement on invasive management of acute coronary syndromes during the COVID-19 pandemic. *Eur. Heart J.* 41 1839–1851. 10.1093/eurheartj/ehaa381 32405641 PMC7239193

[B10] Clavario P. De Marzo V. Lotti R. Barbara C. Porcile A. Russo C. (2020). Assessment of functional capacity with cardiopulmonary exercise testing in non-severe COVID-19 patients at three months follow-up. *medRxiv [preprint]* 10.1101/2020.11.15.20231985

[B11] Clerkin K. J. Fried J. A. Raikhelkar J. Sayer G. Griffin J. M. Masoumi A. (2020). COVID-19 and cardiovascular disease. *Circulation* 141 1648–1655. 10.1161/CIRCULATIONAHA.120.046941 32200663

[B12] Davido B. Seang S. Tubiana R. de Truchis P. (2020). Post-COVID-19 chronic symptoms: a postinfectious entity? *Clin. Microbiol. Infect.* 26 1448–1449. 10.1016/j.cmi.2020.07.028 32712242 PMC7376333

[B13] Desai S. R. Wells A. U. Rubens M. B. Evans T. W. Hansell D. M. (1999). Acute respiratory distress syndrome: CT abnormalities at long-term follow-up. *Radiology* 210 29–35. 10.1148/radiology.210.1.r99ja2629 9885583

[B14] Driggin E. Madhavan M. V. Bikdeli B. Chuich T. Laracy J. Biondi-Zoccai G. (2020). Cardiovascular considerations for patients, health care workers, and health systems during the COVID-19 Pandemic. *J. Am. Coll. Cardiol.* 75 2352–2371. 10.1016/j.jacc.2020.03.031 32201335 PMC7198856

[B15] Evans P. C. Rainger G. Mason J. C. Guzik T. J. Osto E. Stamataki Z., et al. (eds) (2020). Endothelial dysfunction in COVID-19: a position paper of the ESC working group for atherosclerosis and vascular biology, and the esc council of basic cardiovascular science. *Cardiovasc. Res.* 116 2177–2184. 10.1093/cvr/cvaa230 32750108 PMC7454368

[B16] Frija-Masson J. Debray M. P. Gilbert M. Lescure F. X. Travert F. Borie R. (2020). Functional characteristics of patients with SARS-CoV-2 pneumonia at 30 days post-infection. *Eur. Respir. J.* 56:2001754. 10.1183/13993003.01754-2020 32554533 PMC7301832

[B17] Gardner W. N. (1994). “Hyperventilation: diagnosis and therapy,” in *Behavioral and Psychological Approaches to Breathing Disorders*, eds Beverly H. Timmons R. L. (New York: Springer Science + Business Media, LLC), 99–111. 10.1007/978-1-4757-9383-3_7

[B18] Garrigues E. Janvier P. Kherabi Y. Le Bot A. Hamon A. Gouze H. (2020). Post-discharge persistent symptoms and health-related quality of life after hospitalization for COVID-19. *J. Infect.* 81 e4–e6. 10.1016/j.jinf.2020.08.029 32853602 PMC7445491

[B19] Goërtz Y. M. J. Van Herck M. Delbressine J. M. Vaes A. W. Meys R. Machado F. V. C. (2020). Persistent symptoms 3 months after a SARS-CoV-2 infection: the post-COVID-19 syndrome? *ERJ Open Res.* 6 00542–2020. 10.1183/23120541.00542-2020 33257910 PMC7491255

[B20] Huang C. Wang Y. Li X. Ren L. Zhao J. Hu Y. (2020). Clinical features of patients infected with 2019 novel coronavirus in Wuhan. *China Lancet* 395 497–506. 10.1016/S0140-6736(20)30183-5 31986264 PMC7159299

[B21] Huang L. Zhao P. Tang D. Zhu T. Han R. Zhan C. (2020). Cardiac involvement in patients recovered from COVID-2019 identified using magnetic resonance imaging. *JACC Cardiovasc. Imaging* 13 2330–2339. 10.1016/j.jcmg.2020.05.004 32763118 PMC7214335

[B22] Huang Y. Tan C. Wu J. Chen M. Wang Z. Luo L. (2020). Impact of coronavirus disease 2019 on pulmonary function in early convalescence phase. *Respir. Res.* 21:163. 10.1186/s12931-020-01429-6 32600344 PMC7323373

[B23] Hui D. S. Joynt G. M. Wong K. T. Gomersall C. D. Li T. S. Antonio G. (2005). Impact of severe acute respiratory syndrome (SARS) on pulmonary function, functional capacity and quality of life in a cohort of survivors. *Thorax* 60 401–409. 10.1136/thx.2004.030205 15860716 PMC1758905

[B24] Jack S. Rossiter H. B. Warburton C. J. Whipp B. J. (2003). Behavioral influences and physiological indices of ventilatory control in subjects with idiopathic hyperventilation. *Behav. Modif.* 27 637–652. 10.1177/0145445503256318 14531159

[B25] Laffey J. G. Kavanagh B. P. (2002). Hypocapnia. *N. Engl. J. Med.* 347 43–53. 10.1056/NEJMra012457 12097540

[B26] Li T. S. Gomersall C. D. Joynt G. M. Chan D. P. Leung P. Hui D. S. (2006). Long-term outcome of acute respiratory distress syndrome caused by severe acute respiratory syndrome (SARS): an observational study. *Crit. Care Med.* 8 302–308. 17227266

[B27] Lum L. C. (1975). Hyperventilation: the tip and the iceberg. *J. Psychosom. Res.* 19 375–383. 10.1016/0022-3999(75)90017-3 1214233

[B28] Mart M. F. Ware L. B. (2020). The long-lasting effects of the acute respiratory distress syndrome. *Exp. Rev. Respir. Med.* 14 577–586. 10.1080/17476348.2020.1743182 32168460 PMC7454121

[B29] Miglis M. G. Goodman B. P. Chemali K. R. Stiles L. (2020a). Re: ‘post-COVID-19 chronic symptoms’ by Davido et al. *Clin. Microbiol. Infect.* 10.1016/j.cmi.2020.08.028 [Epub ahead of print]. 32891765 PMC7470728

[B30] Miglis M. G. Prieto T. Shaik R. Muppidi S. Sinn D. I. Jaradeh S. (2020b). A case report of postural tachycardia syndrome after COVID-19. *Clin. Auton. Res.* 10.1007/s10286-020-00727-9 [Epub ahead of print]. 32880754 PMC7471493

[B31] Mo X. Jian W. Su Z. Chen M. Peng H. Peng P. (2020). Abnormal pulmonary function in COVID-19 patients at time of hospital discharge. *Eur. Respir. J.* 55:2001217. 10.1183/13993003.01217-2020 32381497 PMC7236826

[B32] Ngai J. C. Ko F. W. Ng S. S. To K. W. Tong M. Hui D. S. (2010). The long-term impact of severe acute respiratory syndrome on pulmonary function, exercise capacity and health status. *Respirology* 15 543–550. 10.1111/j.1440-1843.2010.01720.x 20337995 PMC7192220

[B33] O’Keefe J. B. Tong E. J. Datoo O. Keefe G. A. Tong D. C. (2020). Predictors of disease duration and symptom course of outpatients with acute COVID-19: a retrospective cohort study. *medRxiv [preprint]* 10.1101/2020.06.05.20123471

[B34] Ong K. C. Ng A. W. Lee L. S. Kaw G. Kwek S. K. Leow M. K. (2004). Pulmonary function and exercise capacity in survivors of severe acute respiratory syndrome. *Eur. Respir. J.* 24 436–442. 10.1183/09031936.04.00007104 15358703

[B35] Park W. B. Jun K. I. Kim G. Choi J. P. Rhee J. Y. Cheon S. (2018). Correlation between pneumonia severity and pulmonary complications in middle east respiratory syndrome. *J. Korean Med. Sci.* 33:e169. 10.3346/jkms.2018.33.e169 29892209 PMC5990444

[B36] Pascarella G. Strumia A. Piliego C. Bruno F. Del Buono R. Costa F. (2020). COVID-19 diagnosis and management: a comprehensive review. *J. Intern. Med.* 288 192–206. 10.1111/joim.13091 32348588 PMC7267177

[B37] Puntmann V. O. Carerj M. L. Wieters I. Fahim M. Arendt C. Hoffmann J. (2020). Outcomes of cardiovascular magnetic resonance imaging in patients recently recovered from coronavirus disease 2019 (COVID-19). *JAMA Cardiol.* 5 1265–1273. 10.1001/jamacardio.2020.3557 32730619 PMC7385689

[B38] Raman B. Cassar M. P. Tunnicliffe E. M. Filippini N. Griffanti L. Alfaro-Almagro F. (2020). Medium-term effects of SARS-CoV-2 infection on multiple vital organs, exercise capacity, cognition, quality of life and mental health, post-hospital discharge. *medRxiv [preprint]* 10.1101/2020.10.15.20205054 PMC7808914 33490928

[B39] Rodriguez-Morales A. J. Cardona-Ospina J. A. Gutierrez-Ocampo E. Villamizar-Pena R. Holguin-Rivera Y. Escalera-Antezana J. P. (2020). Clinical, laboratory and imaging features of COVID-19: a systematic review and meta-analysis. *Travel Med. Infect. Dis.* 34:101623. 10.1016/j.tmaid.2020.101623 32179124 PMC7102608

[B40] Schaller T. Hirschbuhl K. Burkhardt K. Braun G. Trepel M. Markl B. (2020). Postmortem examination of patients with COVID-19. *JAMA* 323 2518–2520. 10.1001/jama.2020.8907 32437497 PMC7243161

[B41] Simpson R. Robinson L. (2020). Rehabilitation after critical illness in people with COVID-19 infection. *Am. J. Phys. Med. Rehabil.* 99 470–474. 10.1097/PHM.0000000000001480 32282359 PMC7253039

[B42] Tenforde M. W. Kim S. S. Lindsell C. J. Billig Rose E. Shapiro N. I. Files D. C. (2020). Symptom duration and risk factors for delayed return to usual health among outpatients with COVID-19 in a multistate health care systems network - united states, march-june. *MMWR Morb. Mortal. Wkly. Rep.* 69 993–998. 10.15585/mmwr.mm6930e1 32730238 PMC7392393

[B43] van Dixhoorn J. Duivenvoorden H. J. (1985). Efficacy of nijmegen questionnaire in recognition of the hyperventilation syndrome. *J. Psychosom. Res.* 29 199–206. 10.1016/0022-3999(85)90042-x 4009520

[B44] van Gassel R. J. J. Bels J. L. M. Raafs A. van Bussel B. C. T. van de Poll M. C. G. Simons S. O. (2020). High Prevalence of Pulmonary Sequelae at 3 Months After Hospital Discharge in Mechanically Ventilated COVID-19 Survivors. *Am. J. Respir. Crit. Care Med.* 10.1164/rccm.202010-3823LE Online ahead of print. 33326353 PMC7874313

[B45] Varga Z. Flammer A. J. Steiger P. Haberecker M. Andermatt R. Zinkernagel A. S. (2020). Endothelial cell infection and endotheliitis in COVID-19. *Lancet* 395 1417–1418. 10.1016/s0140-6736(20)30937-5 32325026 PMC7172722

[B46] Weerahandi H. Hochman K. A. Simon E. Blaum C. Chodosh J. Duan E. (2020). Post-discharge health status and symptoms in patients with severe COVID-19. *medRxiv [preprint]* 10.1101/2020.08.11.20172742 33443703 PMC7808113

[B47] Wong A. W. Shah A. S. Johnston J. C. Carlsten C. Ryerson C. J. (2020). Patient-reported outcome measures after COVID-19: a prospective cohort study. *Eur. Respir. J.* 56:2003276. 10.1183/13993003.03276-2020 33008936 PMC7530908

[B48] Zhang P. Li J. Liu H. Han N. Ju J. Kou Y. (2020). Long-term bone and lung consequences associated with hospital-acquired severe acute respiratory syndrome: a 15-year follow-up from a prospective cohort study. *Bone Res.* 8:8. 10.1038/s41413-020-0084-5 32128276 PMC7018717

[B49] Zhao Y. M. Shang Y. M. Song W. B. Li Q. Q. Xie H. Xu Q. F. (2020). Follow-up study of the pulmonary function and related physiological characteristics of COVID-19 survivors three months after recovery. *E Clin. Med.* 25:100463. 10.1016/j.eclinm.2020.100463 32838236 PMC7361108

[B50] Zhou C. Wu L. Ni F. Ji W. Wu J. Zhang H. (2014). Critical illness polyneuropathy and myopathy: a systematic review. *Neural Regen. Res.* 9 101–110. 10.4103/1673-5374.125337 25206749 PMC4146320

